# Supplementary material for: Structural basis of homo- and heterotrimerization of collagen I
Source: Nat Commun. 2017 Mar 10;8:14671. doi: 10.1038/ncomms14671 (PMC5353611; doi:10.1038/ncomms14671)
Supplement: Supplementary Information — Supplementary Figures and Supplementary Table. [file ncomms14671-s1.pdf]

Supplementary Table 1. Details of interactions at the inter-chain interfaces in the crystal structures of homo-CPI and CPIII.

Homo-CPI								
INTERFACE 1 (503.6 A^2)			INTERFACE 2 (538.4 A^2)			INTERFACE 3 (520.0 A^2)		
Chain B	Dist. [Å]	Chain C	Chain F	Dist. [Å]	Chain B	Chain C	Dist. [Å]	Chain F
**S-S bonds**								
CYS 47 [SG]	2.05	CYS 64 [SG]	CYS 47 [SG]	2.09	CYS 64 [SG]	CYS 47 [SG]	2.08	CYS 64 [SG]
**H bonds**								
ARG 39 [NH1]	3.33	ASN 61 [O]	ARG 39 [NH1]	2.68	ASN 61 [O]	ARG 39 [NH1]	2.93	ASN 61 [O]
ARG 39 [NH1]	3.35	GLN 62 [OE1]	ASP 43 [OD1]	2.90	CYS 64 [N]	ARG 39 [NH1]	3.62	GLN 62 [OE1]
ASP 43 [OD1]	2.83	CYS 64 [N]	GLN 62 [NE2]	3.00	GLN 62 [OE1]	ASP 43 [OD1]	2.94	CYS 64 [N]
MET 46 [SD]	3.64	ASP 67 [N]				MET 46 [SD]	3.64	ASP 67 [N]
						GLN 62 [NE2]	3.30	GLN 62 [OE1]
**Salt bridges**								
ARG 42 [NH1]	3.25	ASP 67 [OD2]	ARG 42 [NH1]	3.34	ASP 67 [OD2]	ARG 42 [NH1]	3.17	ASP 67 [OD2]
ARG 42 [NH1]	2.85	ASP 129 [OD1]	ARG 42 [NH2]	2.85	ASP 67 [OD2]	ARG 42 [NH2]	2.94	ASP 67 [OD2]
ARG 42 [NH2]	3.25	ASP 67 [OD2]	ARG 42 [NE]	3.95	ASP 129 [OD1]	ARG 42 [NH1]	2.67	ASP 129 [OD1]
			ARG 42 [NH2]	2.86	ASP 129 [OD1]			

CPIII								
INTERFACE 1 (740.3 A^2)			INTERFACE 2 (652.5 A^2)			INTERFACE 3 (514.9 A^2)		
Chain A	Dist. [Å]	Chain B	Chain B	Dist. [Å]	Chain C	Chain C	Dist. [Å]	Chain A
**S-S bonds**								
CYS 47 [SG]	2.03	CYS 64 [SG]	CYS 47 [SG]	2.04	CYS 64 [SG]	CYS 47 [SG]	2.04	CYS 64 [SG]
**H bonds**								
ASP 43 [OD2]	3.30	CYS 64 [N]	ASP 43 [OD2]	3.16	CYS 64 [N]	ARG 39 [NH1]	2.83	ASN 61 [O]
SER 141 [OG]	2.91	ASP 130 [OD2]	SER 141 [OG]	3.15	ASP 130 [OD2]	ASP 43 [OD2]	3.01	CYS 64 [N]
LYS 214 [NZ]	2.93	SER 174 [O]				SER 141 [OG]	3.16	ASP 127 [O]
						SER 141 [OG]	3.20	ASP 130 [OD2]
**Salt bridges**								
ARG 42 [NE]	2.93	ASP 127 [OD2]	ARG 42 [NE]	2.96	ASP 127 [OD2]	ARG 142 [NH1]	3.85	ASP 127 [OD1]
ARG 142 [NH2]	3.31	GLU 126 [OE2]	ARG 213 [NH2]	3.51	ASP 130 [OD2]	ARG 142 [NH1]	3.00	ASP 127 [OD2]
ARG 142 [NH2]	4.00	ASP 130 [OD2]						
LYS 186 [NZ]	3.06	GLU 176 [OE1]						
ARG 217 [NH2]	3.36	GLU 176 [OE1]						
ARG 217 [NH2]	3.28	GLU 176 [OE2]						

Conserved/homologous residues in the base region are highlighted in red, with conserved/homologous residues in the petal region highlighted in blue. Residues in the petal region that are specific to each procollagen type are highlighted in purple. There are no such specific residues in the base region for either homo-CPI or CPIII. The interfaces for homo-CPI do not include the coiled-coil region as this was absent from the structure used to calculate the interfaces for CPIII (PDB code 4AE2). Data for only one of the two trimers in the asymmetric unit are shown for homo-CPI, interactions for the second trimer being very similar. Individual chains in homo-CPI were automatically identified as B, C and F during structure determination, with A, D and E being used for the other trimer in the asymmetric unit.

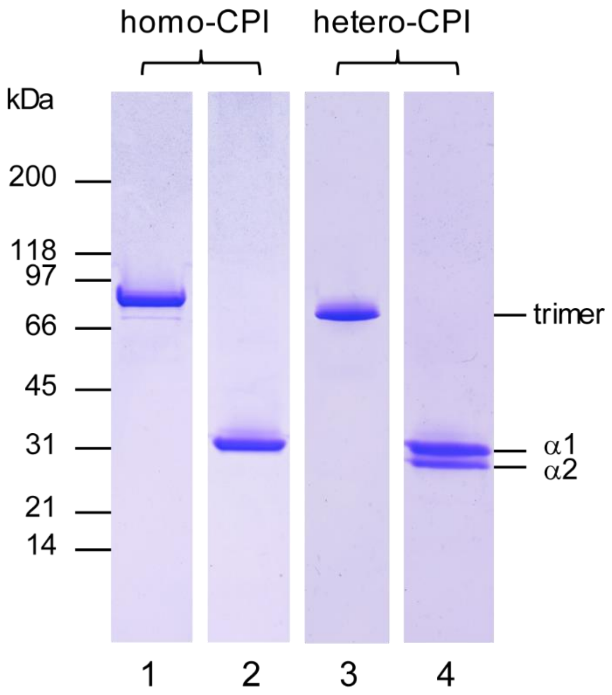

Supplementary Figure 1. Purification of recombinant forms of homo-CPI and hetero-CPI. Samples from the final gel filtration step are shown. Track 1: homo-CPI, non-reducing conditions; track 2: homo-CPI, reducing conditions; track 3: hetero-CPI, non-reducing conditions; track 4: hetero-CPI, reducing conditions. SDS-PAGE (4-20 % acrylamide gradient gels), staining with Coomassie Blue. For hetero-CPI, the N-terminal His-tag on the α2(I) chain has been removed by cleavage with TEV protease. Data shown are representative of triplicate (A) biological replicates.

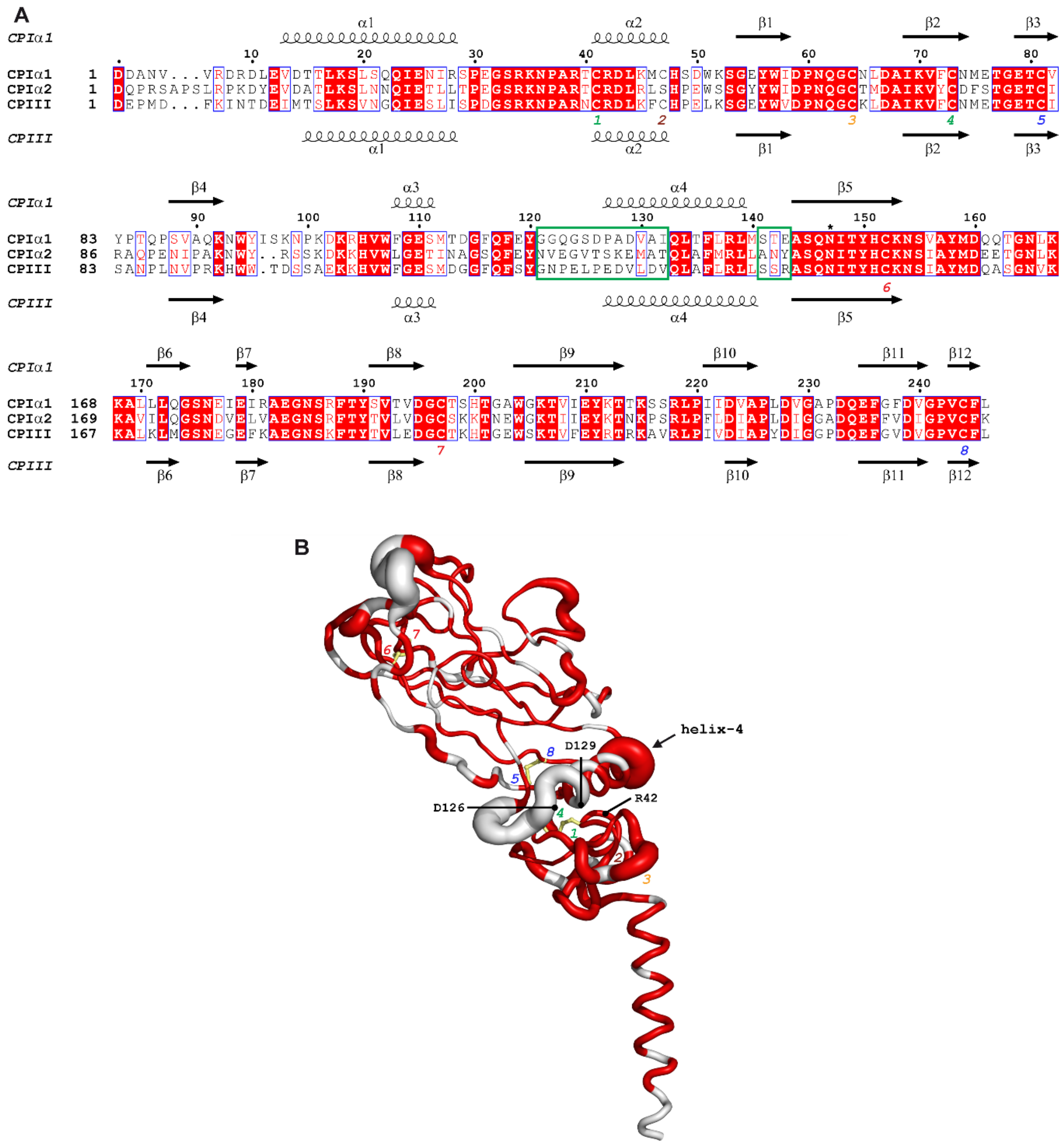

Supplementary Figure 2. Sequence and structural alignments of CPI and CPIII. (A) Sequence alignments, performed using *ESPript*, with corresponding secondary structures based on the crystal structures of homo-CPI and CPIII. Identical residues are indicated by white labels on a red background, conservative changes by red on white and non-conserved changes by black on white. Blue boxes indicate identical/conserved regions. Numbering, based on the CPI α1 chain, begins after the BMP-1 cleavage site; there are differences in residue numbering, due to insertions and deletions, for CPIα2 and CPIII. The long and short stretches of the CRS are outlined in green. In the constructs used here, the Asn residue in the N-glycosylation site (indicated by *) was mutated to Gln. Cysteine residues involved in intra-chain disulphide bonds (1-4, 5-8, 6-7) are numbered as pairs with the same colour, while those involved in inter-chain disulphide bonds (2, 3) are in different colours. (B) Structural alignment between one chain from homo-CPI and one chain from CPIII (PDB code 4AK3), obtained using *ENDscript*. Sequence similarity is indicated by the colour, where red indicates identical residues and grey non-identical. The width of the ribbon is proportional to the spatial separation between corresponding residues in the aligned structures. Positions of cysteine residues are indicated (same colour code as in A), as well as helix 4 and positions of key residues in CPIα1 (R42, D126, D129). Note that in this orientation R42 is at the back with D126 and D129 at the front.

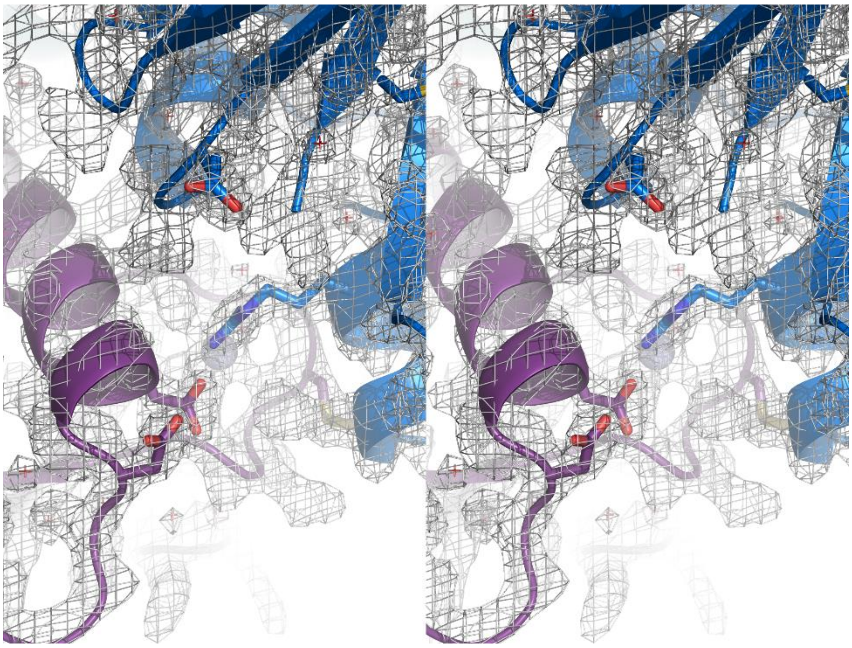

Supplementary Figure 3. Portion of the electron density map. Stereo drawing showing the 2Fo-Fc electron density contoured at 1.6σ around charged residues at the inter-chain interface in homo-CPI. Colouring as in Fig. 3B.

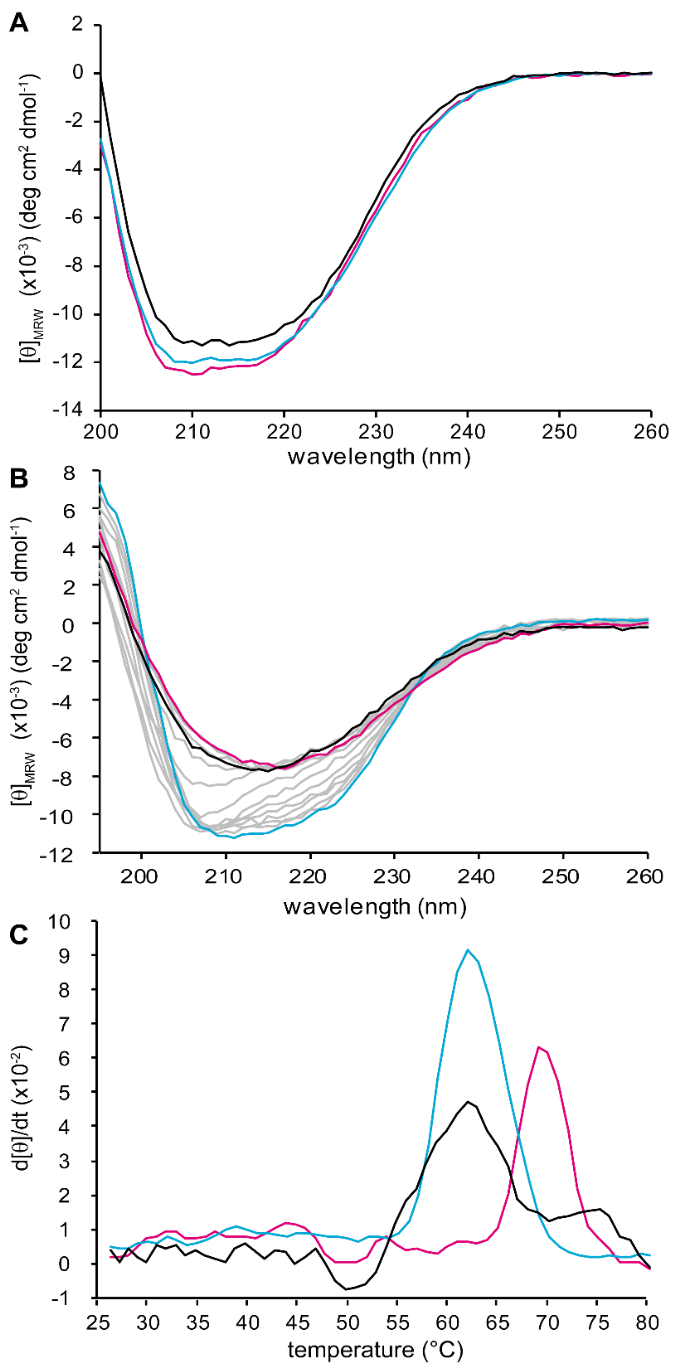

Supplementary Figure 4. Circular dichroism analysis of C-propeptide trimers. (A) Far UV spectra for His-tagged CPIII (black), His-tagged homo-CPI (magenta) and non-tagged hetero-CPI (turquoise), measured at 25 °C. The curves show mean residue molar ellipticities (MRME) in the wavelength range 200 nm to 260 nm. (B) Far UV spectra for CPIII measured in the temperature range 25 °C (turquoise) to 85 °C (magenta), in steps of 5 °C, and after cooling to 25 °C (black). The curves show MRME values at each temperature in the wavelength range 195 nm to 260 nm, with data for intermediate temperatures in grey. (C) Temperature dependence of the MRME data for CPIII (black), His-tagged homo-CPI (magenta) and non-tagged hetero-CPI (turquoise) measured at 208 nm in the range 25 °C to 80 °C. The curves show the first derivative of the MRME versus temperature curves (where the peaks correspond to the mid-points in the thermal transitions). MRW = mean residue weight. Data shown are representative of triplicate technical replicates.

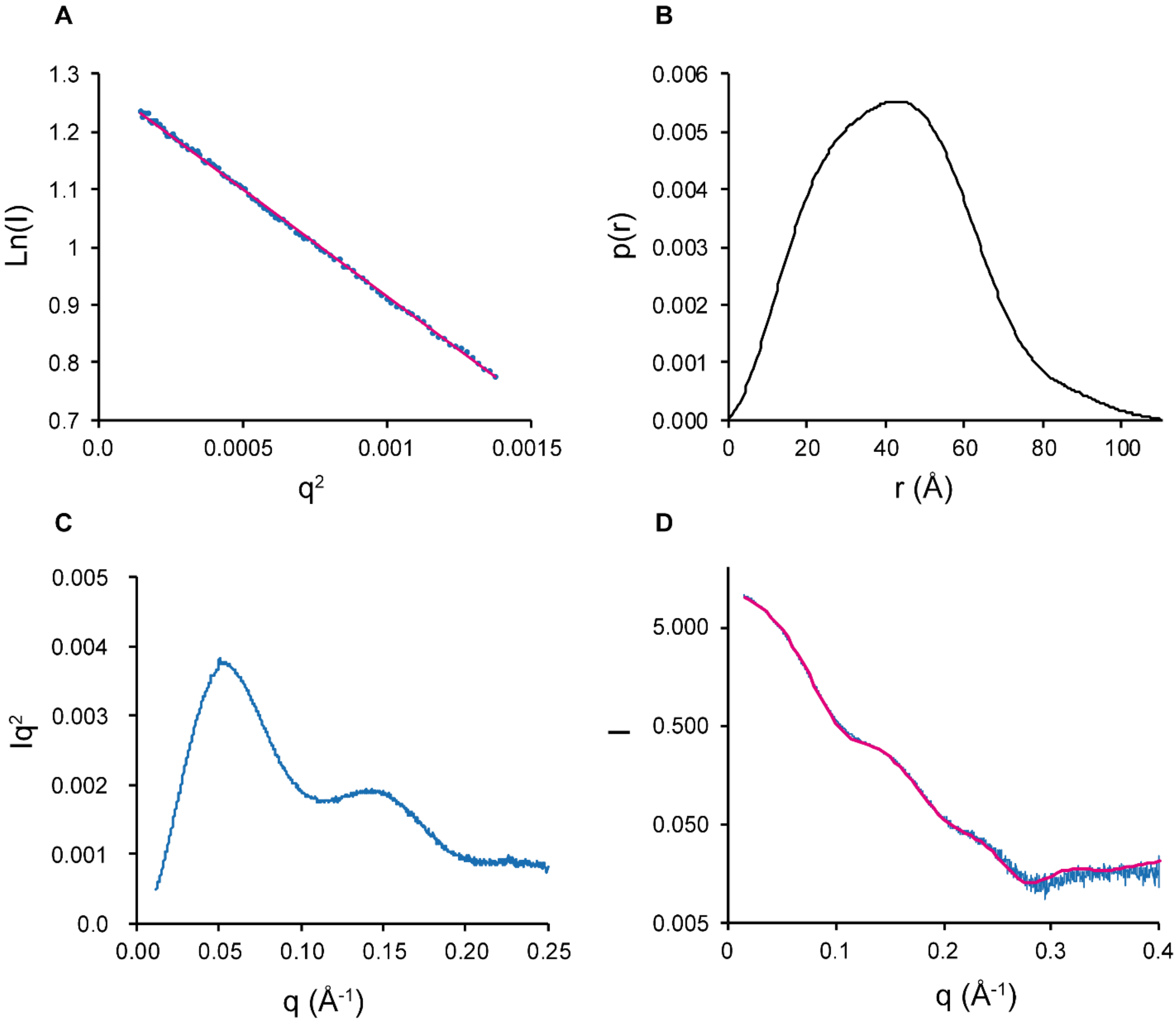

Supplementary Figure 5. Small angle X-ray scattering of hetero-CPI. (A) Linear Guinier plot corresponding to a radius of gyration of 33.4 Å. Experimental data in blue. (B) Distance distribution function p(r) showing a maximum dimension of approximately 110 Å. (C) Kratky curve calculated from the scattering data indicating a well-folded multidomain protein. (D) Comparison of the observed angular dependence of the SAXS data for His-tagged hetero-CPI (in blue) with the theoretical curve (in magenta) calculated using *WAXSiS* based on the crystal structure of homo-CPI.

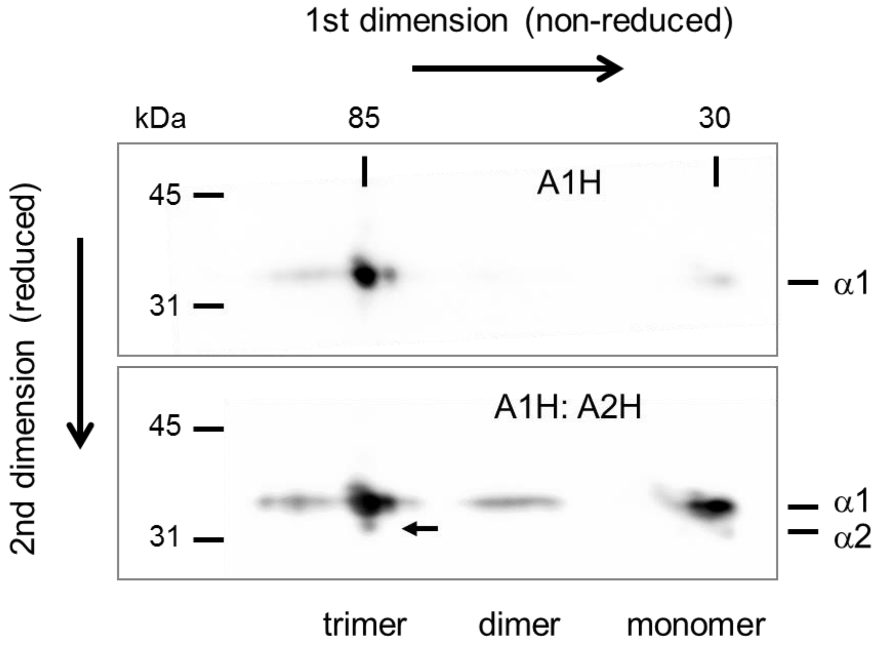

Supplementary Figure 6. Composition of trimers analysed by two-dimensional SDS-PAGE and Western blotting. Since non-reducing gels of conditioned medium sometimes showed the presence of monomers (and other forms) in addition to the trimers migrating at ~85kDa, gels (10 % acrylamide) were run first in non-reducing conditions then individual tracks were cut out, reduced and alkylated, placed horizontally on a second gel (one for each track) and run in a second dimension (reducing conditions) followed by Western blotting using anti-His tag antibodies. Unlike the homo-CPI trimer (A1H), which migrates as a single band in reducing conditions, the hetero-CPI trimer (A1H:A2H) is resolved into two bands, corresponding to the α1 and α2 chains, the latter indicated by an arrow. Since the α1:α2 ratio is clearly much greater than 2:1, this shows that the band migrating at 85 kDa consists of both homotrimers and heterotrimers. Data shown are representative of triplicate biological replicates.

```
                                   10       20        30          40        50            60        70         80
                                    .        .         .           .         .             .         .          .
P02452_CO1A1_HUMAN_1228-1464   *10  DLEVDTTLKSLSQQIENIRSPEG-SRKNPARTCRDLKMCHSDW----KSGEYWIDPNQGCNLDAIKVFCNMET-GETCVYPT   85
P08123_CO1A2_HUMAN_1132-1366   *13  DYEVDATLKSLNNQIETLLTPEG-SRKNPARTCRDLRLSHPEW----SSGYYWIDPNQGCTMDAIKVYCDFST-GETCIRAQ   88
P02458_CO2A1_HUMAN_1252-1487   *11  DAEVDATLKSLNNQIESIRSPEG-SRKNPARTCRDLKLCHPEW----KSGDYWIDPNQGCTLDAMKVFCNMET-GETCVYPN   86
P02461_CO3A1_HUMAN_1231-1466   *10  TDEIMTSLKSVNGQIESLISPDG-SRKNPARNCRDLKFCHPEL----KSGEYWVDPNQGCKLDAIKVFCNMET-GETCISAN   85
P05997_CO5A2_HUMAN_1265-1499   *12  DPGVHATLKSLSSQIETMRSPDG-SKKHPARTCDDLKLCHSAK----QSGEYWIDPNQGSVEDAIKVYCNMET-GETCISAN   87
A0A084WGV4_ANOSI_1342-1565       1  QKLVENAYEKLKSAFATFKKPDG-KQGSPAKTCRDLFAAHPEF----TSGNYWIDPNEGDARDAILVYCDAEK-KASCVLPQ   76
A0A0A1X7I7_BACCU_1333-1557       1  EAMVIKAFEHLKASFERLRRPNG-QQSAPAKTCRDLFAAYPDY----KSGEYWIDPNEADPRDAILVYCDRET-RGSCILPK   76
A0A087ZYL7_APIME_1278-1501       1  QELIQKAYKQLKSSFQKFIKPDG-EKNSPAKTCRDLYSAYPNK----LSGEYWIDPNEGDARDAILVYCDAKK-RATCLLPN   76
A0A0P6AH74_9CRUS_1341-1565       1  KALVVKAYEQLKVSFDKYTKPSG-DKAAPARTCRDLAVAHPEL----PSADYWIDPNQGDTKDSILVFCDMNR-RATCIRPK   76
A0A0A1X8A9_BACCU_1680-1904       1  -VDMYSAIYSMRLEMDRMRKPTG-TQDNPVRTCRDLHYAHPQF----ENGWYWVDPNAGMPDDAIFVYCNMSAGGETCIQPD   76
A0A088AKC6_APIME_1532-1755       1  -LDMYSSIYAMRQELDRIRKPIG-SRENPARTCKDLFYGHPHF----HDGWYWIDPNLGMADDSVYVYCNMTNMGETCVYPD   76
A0A084VDT1_ANOSI_1490-1715       1  -LDMYSSIYSMRQELDRIRKPVG-TRENPARTCRDLHHGHPQF----KDGWYWIDPNLGMGDDAVYVFCNMTAEGETCVYPD   76
A0A0P4WM94_9CRUS_1469-1693       1  -LDMYSSIYTMRQDLERIKKPQG-SKENPVRSCKDLYFGHPQF----KDGWYWIDPNLGMPDDAIYVFCNMTGSGETCVYPD   76
P12107_COBA1_HUMAN_1576-1806   *11  MEEIFGSLNSLKQDIEHMKFPMG-TQTNPARTCKDLQLSHPDF----PDGEYWIDPNQGCSGDSFKVYCNFTSGGETCIYPD   87
P20908_CO5A1_HUMAN_1608-1838   *14  MEEIFGSLNSLKLEIEQMKRPLG-TQQNPARTCKDLQLCHPDF----PDGEYWVDPNQGCSRDSFKVYCNFTAGGSTCVFPD   90
P25940_CO5A3_HUMAN_1513-1745   *13  LEEVLASLTSLSLELEQLRRPPG-TAERPGLVCHELHRNHPHL----PDGEYWIDPNQGCARDSFRVFCNFTAGGETCLYPD   89
P13942_COBA2_HUMAN_1540-1736   *16  LEEIFGSLDSLREEIEQMRRPTG-TQDSPARTCQDLKLCHPEL----PDGEYWVDPNQGCARDAFRVFCNFTAGGETCVTPR   92
Q8IZC6_CORA1_HUMAN_1659-1860     1  GGEIFKTLHYLSNLIQSIKTPLG-TKENPARVCRDLMDCEQKM----VDGTYWVDPNLGCSSDTIEVSCNFTHGGQTCLKPI   77
Q17RW2_COOA1_HUMAN_1514-1714     1  SEEIFKTLNYLSNLLHSIKNPLG-TRDNPARICKDLLNCEQKV----SDGKYWIDPNLGCPSDAIEVFCNFSAGGQTCLPPV   77
H2YGA7_CIOSA_1140-1366           1  -EEIYAAMETLKQELEMMKEPMGRTQDNPGRSCKDIWLCHPDF----PSGNYWIDPNGGCSADAIEVFCDFEAEGDTCISPV   77
H2YJN4_CIOSA_1188-1414           1  -PEMMLVLKELTSSVEDIKAPRGVSRKTPARSCLDIYLAEQQQGTVPKSGVRWIDPNGGCNADGLEVYCNFHT-METCVYPT   80
Cysteine positions:                                                 1     2                    3        4        5
Secondary structure:                   -------α1-------             --α2---         -β1-            --β2--     -β3-
Conservation:                          5      6         9 9     966 9 87   66       68 897999 8   95  9697    5 795 7
```

Supplementary Figure 7 (in three parts). Alignments of human, arthropod and ascidian fibrillar procollagen C-termini. Each sequence is identified by an access code for the full-length protein followed by the start and end positions of the region selected. Human sequences are indicated by collagen type (3 characters) and chain number (2 characters; e.g. CO5A2 = α2 chain of procollagen V) where COB corresponds to procollagen XI, COO to collagen XXIV and COR to collagen XXVII. Abbreviations for other species are as follows: ANOSI = *Anopheles sinensis* (insecta, mosquito); BACCU = *Bactrocera cucurbitae* (insecta, melon fly); APIME = *Apis mellifera* (insecta, honeybee); 9CRUS = *Daphnia magna* (crustacea, water flea); CIOSA = *Ciona savignyi* (ascidiacea, sea squirt). Structure-based sequence alignment was done with the programme PROMALS3D using the 3D structures of the homo-CPI chains as templates. Numbering at the top refers to the CO1A1 chain. Cysteines are highlighted (yellow) and their positions identified at the bottom as in Supplementary Fig. 2. Bold underlined characters in red (negatively charged) or blue (positively charged) show residues in CPI and CPIII involved in inter-chain salt bridge interactions. Regions of secondary structure are shown at the bottom as is the conservation score (maximum 9) for each position. For each sequence, start (left) and end (right) positions are numbered either from known or putative C-propeptide cleavage sites (*) or from the start of the COLFI domain as defined by UniProt, which is always C-terminal to the cleavage site. Outlined in blue (in part 2) are the long and short stretches of the CRS, and in orange the 6/7 residue sequence found only in chordates.

```
                                      90        100       110       120       130       140       150       160
                                       .         .         .         .         .         .         .         .
P02452_CO1A1_HUMAN_1228-1464  *86  QPSVAQKNWYISKNPKDKRHVWFGESMTDGFQFEYGGQGSDPADVAIQLTFLRLMSTEASQNITYHCKNSVAYMDQQTGN-LKKA  169
P08123_CO1A2_HUMAN_1132-1366  *89  PENIPAKNWYRS--SKDKKHVWLGETINAGSQFEYNVEGVTSKEMATQLAFMRLLANYASQNITYHCKNSIAYMDEETGN-LKKA  170
P02458_CO2A1_HUMAN_1252-1487  *87  PANVPKKNWWSSKSK-EKKHIWFGETINGGFHFSYGDDNLAPNTANVQMTFLRLLSTEGSQNITYHCKNSIAYLDEAAGN-LKKA  169
P02461_CO3A1_HUMAN_1231-1466  *86  PLNVPRKHWWTDSSA-EKKHVWFGESMDGGFQFSYGNPELPEDVLDVHLAFLRLLSSRASQNITYHCKNSIAYMDQASGN-VKKA  168
P05997_CO5A2_HUMAN_1265-1499  *88  PSSVPRKTWWASKSP-DNKPVWYGLDMNRGSQFAYGDH-QSPNTAITQMTFLRLLSKEASQNITYICKNSVGYMDDQAKN-LKKA  169
A0A084WGV4_ANOSI_1342-1565     77  PMRTKELHYDG-----DEQEVWLGELK-DGMKITYK-------SDSNQIGFLQLLSARASQNITYHCKNTVAYFNKATNS-YRQS  147
A0A0A1X7I7_BACCU_1333-1557     77  PQETPNLSYNG-----AERETWLSEMP-GGMKITYK-------TDSHQLGFLQLLSAKATQKITFNCRNTIGYLDADETR-NRNG  147
A0A087ZYL7_APIME_1278-1501     77  PVHSPEIIHIT-----DQPETWLSEIE-NGMKITYK-------ADSNQIGFLQLLSKNAYQNITYHCKNSIGYFDSERKT-YRKG  147
A0A0P6AH74_9CRUS_1341-1565     77  PEKTKQITYLGK----PRAEVWFSEMD-SGFQFTYK-------SDSNQMTFLQLLSTHGSQNLTYHCRNSVANYDANDRS-FKKS  148
A0A0A1X8A9_BACCU_1680-1904     77  AHTAEAPLVPRR---QAGELDWYSRLS-GGEKITYDG------VGTVQLTFLRLLTEEAHQNFTYICSNSVAWYSDAERG-YSKS  150
A0A088AKC6_APIME_1532-1755     77  IHTTQMPNIPWR--KENNKTDWYSNLR-GGFKITYEA------IGVVQLNFLRLLSQEAYQNFTYTCINSVAWYNILNFN-YNSS  151
A0A084VDT1_ANOSI_1490-1715     77  IHSSQMPTIPWR--KENDKTDWYSNLR-GGFRISYET------IGTVQMTFLRLLSQEAYQNFTYACMNSVAWYSTQDES-FDNA  151
A0A0P4WM94_9CRUS_1469-1693     77  LQSSKMPNIPWR-KEVGGKEEWYSNMR-GASKVTYET------VGVVQMTFLRLLSQKAHQNFTFTCVNSAAWYNQRTFN-YDQA  152
P12107_COBA1_HUMAN_1576-1806  *88  KKSEGVRISSWP---KEKPGSWFSEFK-RGKLLSYLDV-EGNSINMVQMTFLKLLTASARQNFTYHCHQSAAWYDVSSGS-YDKA  166
P20908_CO5A1_HUMAN_1608-1838  *91  KKSEGARITSWP---KENPGSWFSEFK-RGKLLSYVDA-EGNPVGVVQMTFLRLLSASAHQNVTYHCYQSVAWQDAATGS-YDKA  169
P25940_CO5A3_HUMAN_1513-1745  *90  KKFEIVKLASWS---KEKPGGWYSTFR-RGKKFSYVDA-DGSPVNVVQLNFLKLLSATARQNFTYSCQNAAAWLDEATGD-YSHS  168
P13942_COBA2_HUMAN_1540-1736  *93  DDVT---------------------------QFSYVDS-EGSPVGVVQLTFLRLLSVSAHQDVSYPCSGAAR----------DGP  139
Q8IZC6_CORA1_HUMAN_1659-1860   78  TAS----------------------------KVEFA-------ISRVQMNFLHLLSSEVTQHITIHCLNMTVWQEGTGQTPAKQA  127
Q17RW2_COOA1_HUMAN_1514-1714   78  SVT----------------------------KLEFG-------VGKVQMNFLHLLSSEATHIITIHCLNTPRWTSTQTSG-PGLP  126
H2YGA7_CIOSA_1140-1366         78  ERTASVSWLTSKRWPKAQPGDWFSSYR-MGDRFEYN-------TSIPQFNFLRLLSSQAKQRFTYKCVNSIGWENQQTGS-FDQA  153
H2YJN4_CIOSA_1188-1414         81  NRNIENGTHYTG----EPGHTYYGEEMTRVEHA----------DYASQLTFLRLLSSKAKQQVTFFCRNMVAYYDASADN-KAQA  150
Cysteine positions:                                                                                  6
Secondary structure:                 --β4-                -α3-              ------α4------   ----β5----
Conservation:                                           857     6    7            8  98 986  6 8  86 9 6   6
```

Supplementary Figure 7 (continued).

```
                                   170       180       190       200       210       220       230       240
                                    .         .         .         .         .         .         .         .
P02452_CO1A1_HUMAN_1228-1464   *170 LLLQGSNEIEIRAEGNSRFTYSVTVDGCTSHTGAWGKTVIEYKTTKTSRLPIIDVAPLDVGAPDQEFGFDVGPVCFL-  246
P08123_CO1A2_HUMAN_1132-1366   *171 VILQGSNDVELVAEGNSRFTYTVLVDGCSKKTNEWGKTIIEYKTNKPSRLPFLDIAPLDIGGADQEFFVDIGPVCFK-  247
P02458_CO2A1_HUMAN_1252-1487   *170 LLIQGSNDVEIRAEGNSRFTYTALKDGCTKHTGKWGKTVIEYRSQKTSRLPIIDIAPMDIGGPEQEFGVDIGPVCFL-  246
P02461_CO3A1_HUMAN_1231-1466   *169 LKLMGSNEGEFKAEGNSKFTYTVLEDGCTKHTGEWSKTVFEYRTRKAVRLPIVDIAPYDIGGPDQEFGVDVGPVCFL-  245
P05997_CO5A2_HUMAN_1265-1499   *170 VVLKGANDLDIKAEGNIRFRYIVLQDTCSKRNGNVGKTVFEYRTQNVARLPIIDLAPVDVGGTDQEFGVEIGPVCFV-  235
A0A084WGV4_ANOSI_1342-1565      148 VKLLAWNDAELTARGPQRLRYEALQDDCQHRTAHYAQSVLSYSTDKPMRLPIIDIAVRDVGESNQQFWVEIGAVCFH-  224
A0A0A1X7I7_BACCU_1333-1557      148 LKLLSWNDAELTPKGPMRLRYVAESDECRHRSNAWAKTVITYKTEKPQRLPIVDVKIRDVGEANQQFRIELGPVCFYT  225
A0A087ZYL7_APIME_1278-1501      148 MKFLTWNDAELTPRGNQRLRYEMIIDECRTHNGKWGKTIISYQTDKTIRLPIIDVALRDIGKPNQSFYIEIGNVCYE-  224
A0A0P6AH74_9CRUS_1341-1565      149 MKILGWNDIELNAMGKKRFKYEVIEDECKSRADTWAKSVITFETDKPNRLPFVDVGIFDIGEPNQQFSLEIGMACFW-  225
A0A0A1X8A9_BACCU_1680-1904      151 LRLLGENEMEIANEGT-DIKPEVLRDECQQPN-QRGETVLLVRTKRHNYLPLVDFYPQDYARTDQAFGFKVGPACFK-  225
A0A088AKC6_APIME_1532-1755      152 IRLLGANEDEFSYTG---IKPQIVMDNCKTRK-NKGETVLLIQSKKLQQLPLVDFYPIDYGLPHQAFGFTVGPICFK-  224
A0A084VDT1_ANOSI_1490-1715      152 LRFLGENEIDIGYEQSK-IKPTVLVDGCKTGR-SKSETVFEIRTPKLQYLPIIDFYPVDYGLPQQAFGFQVGPVCFK-  226
A0A0P4WM94_9CRUS_1469-1693      153 IKLLGDNEQEFSAKG---VRPNVILDGCKNRK-GSSKTVFEIRSDKLGQLPIIDFFPVDYGQPHQAFGFEVGPVCFK-  225
P12107_COBA1_HUMAN_1576-1806   *167 LRFLGSNDEEMSYDNN--PFIKTLYDGCASRK-GYEKTVIEINTPKIDQVPIVDVMINDFGDQNQKFGFEVGPVCFLG  241
P20908_CO5A1_HUMAN_1608-1838   *170 LRFLGSNDEEMSYDNN--PYIRALVDGCATKK-GYQKTVLEIDTPKVEQVPIVDIMFNDFGEASQKFGFEVGPACFMG  244
P25940_CO5A3_HUMAN_1513-1745   *169 ARFLGTNGEELSFNQTTAATVSVPQDGCRLRK-GQTKTLFEFSSSRAGFLPLWDVAATDFGQTNQKFGFELGPVCFSS  245
P13942_COBA2_HUMAN_1540-1736   *140 LRLRGANEDELSPETS--PYVKEFRDGCQT---QQGRTVLEVRTPVLEQLPVLDASFSDLGAPPRRGGVLLGPVCFMG  212
Q8IZC6_CORA1_HUMAN_1659-1860    128 VRFRAWNGQIFEAGGQ--FRPEVSMDGCKVQDGRWHQTLFTFRTQDPQQLPIISVDNLPPASSGKQYRLEVGPACFL-  202
Q17RW2_COOA1_HUMAN_1514-1714    127 IGFKGWNGQIFKVNTL--LEPKVLSDDCKIQDGSWHKATFLFHTQEPNQLPVIEVQKLPHLKTERKYYIDSSSVCFL-  201
H2YGA7_CIOSA_1140-1366          154 IHLLAANDEVLTYGS---EHLTVIEDNCKTGH-GNGQVVLELRTREVDLLPLFDYKAFDFGTRSQRHGYQLDRVCFSG  227
H2YJN4_CIOSA_1188-1414          151 LKLRGFGDAEFTAEGAVGTTYRVLHDGCSTRPTQWDRTEIEFETRLVGRMPITDIAPFDIGDADQQFGAKFGPVCFK-  227
Cysteine positions:                                            7                                              8
Secondary structure:                 -β6-    -β7-        --β8-        ------β9-----     -β10-        --β11- -β12-
Conservation:                         5 5 85 5               9 9         5 5   7     69  6    7 6   6 5    6 698
```

Supplementary Figure 7 (end).

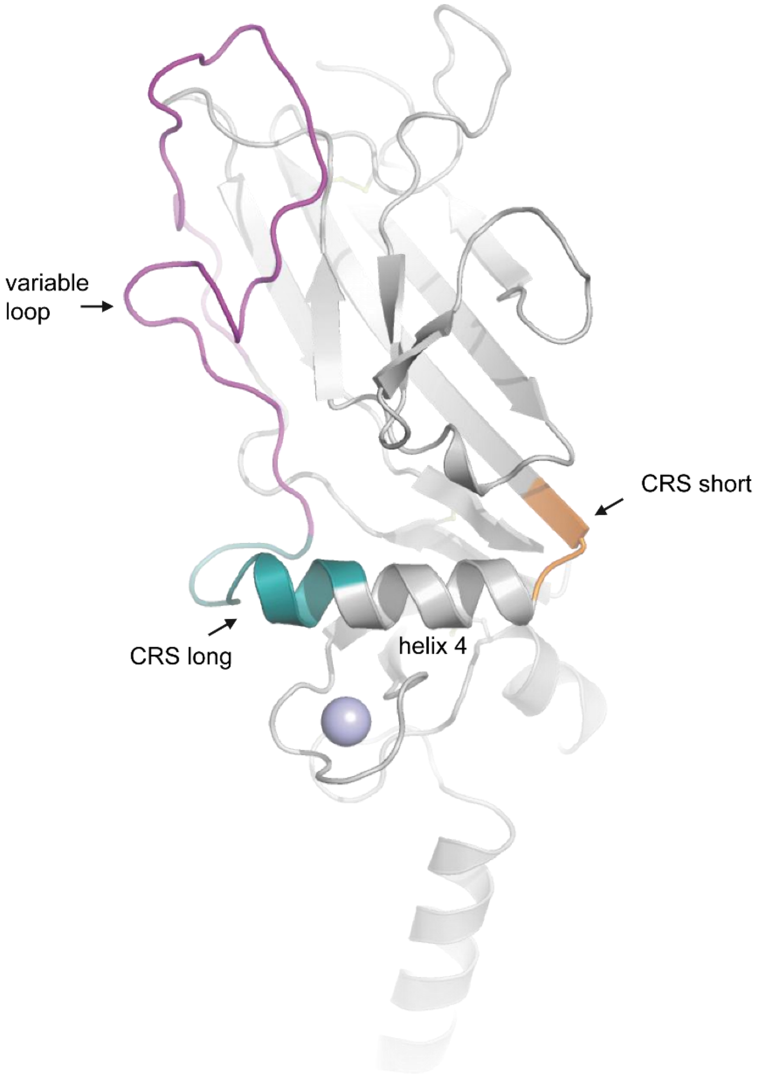

Supplementary Figure 8. Chain recognition sequence and variable loop. Shown is one of the chains from homo-CPI (in grey, bound Ca^{2+} in light blue) with the long and short stretches of the CRS marked in deap teal (blueish green) and light orange, respectively. The relatively poorly conserved ~30 residue sequence that precedes the CRS long (see Supplementary Fig. 5), shown in magenta, is part of a surface-exposed loop in the petal region that is not involved in inter-chain interactions. See also Supplementary Movie 5.
